# A clinical audit of red blood cell transfusion practices at a district hospital in South Africa

**DOI:** 10.4102/safp.v66i1.5958

**Published:** 2024-08-08

**Authors:** Nonofo S. Madito, Cornel van Rooyen, Dirk T. Hagemeister

**Affiliations:** 1 Department of Family Medicine, Faculty of Health Sciences, University of the Free State, Bloemfontein, South Africa; 2 Department of Biostatistics, Faculty of Health Sciences, University of the Free State, Bloemfontein, South Africa

**Keywords:** red blood cells, transfusion, ahemoglobin, guidelines, audit, anaemia

## Abstract

**Background:**

Red blood cell (RBC) transfusion is one of the most critical and expensive lifesaving treatment modalities. A clinical audit is a valuable instrument to determine whether transfusion practices align with the guidelines and identify knowledge deficiencies. The study aimed to evaluate the RBC transfusion practices and patient outcomes at the National District Hospital in Bloemfontein, South Africa, and to determine adherence to transfusion guidelines.

**Methods:**

A retrospective descriptive study was conducted. All blood transfusion registers in the hospital were used to identify transfusion episodes during the study period. Files were retrieved from the admissions office and information captured on a paper-based datasheet. The appropriateness of the transfusion and adherence to the South African transfusion guidelines were evaluated using specific criteria.

**Results:**

Of the 118 transfusion episodes during the study period, 78 files were retrieved and 76 included in the study. The patients’ median age was 47 years (interquartile range [IQR]: 32–66 years), with human immunodeficiency viruses (HIV) (*n* = 34; 44.7%) being the most common comorbid condition. Pre-transfusion haemoglobin was documented for all patients with a median of 4.6 g/dL (IQR: 3.95 g/dL – 5.5 g/dL). The audit revealed that in 68.4% (*n* = 52) of the cases, the guidelines were applied appropriately.

**Conclusion:**

The study described the blood transfusion practices and identified shortcomings when compared with the standard clinical guidelines.

**Contribution:**

The study highlights the importance of applying rationale, caution and consideration of the specific patient profile when performing transfusions.

## Introduction

South Africa is a high-middle-income country with a resource-limited public health sector. Red blood cell (RBC) transfusion is one of the most critical and expensive lifesaving treatment modalities. It thus has to be used correctly and judiciously, considering the risks versus benefits and patient outcomes.^1,2^

More than 118.5 million blood donations are performed globally on an annual basis.^3^ The blood donation pool in South Africa consists of less than 1% of the population, which makes it difficult to reach the daily target of 3300 units, necessitating the need for more prudent use of blood products.^4^ However, the endorsement of what is commonly referred to as the ‘three pillars of patient blood management’ by the World Health Assembly resolution in 2010,^3^ led to decreased transfusion episodes resulting in cost savings and improved patient outcomes.^5,6^

In 2018, an estimated 30.8% of adults in the South African population of 57.8 million had anaemia.^6^ Women of childbearing age and the elderly were severely affected. Common types of anaemia were iron deficiency, human immunodeficiency viruses (HIV)-related anaemia and anaemia because of inflammation.^6^

The high prevalence of HIV in South Africa also reduces the number of potential blood donors and increases morbidity in the general population.^6,7^ Approximately 20% of possible blood donors could not donate because of their HIV-positive status and other transmissible infections. Therefore, anaemia should be managed appropriately to decrease the demand for blood transfusion and improve the population’s general health and productivity.^6^

A study conducted at academic hospitals affiliated to the University of the Witwatersrand, South Africa, investigated the knowledge of blood and blood product usage among doctors of all levels, that is, interns, community service doctors, medical officers and consultants, in the Department of Internal Medicine.^7^ The knowledge areas that were evaluated included RBC usage, consent, ordering of blood products, adverse effects of blood products and platelet transfusions. The findings revealed that their knowledge was generally deficient even though consultants performed better than their juniors. The following average scores were obtained for the above mentioned knowledge areas. Interns scored the lowest at 56%, community service doctors scored 58%, medical officers and registrars scored 63%, while consultants had the highest score of 64%.^7^

In a similar study among oncologists in Uganda,^8^ most doctors admitted having knowledge gaps and needing more training in transfusion medicine. Their decision to transfuse was reportedly often based on peer influence, leading to inappropriate transfusions, to which the absence of transfusion guidelines further contributed.^8^

A clinical audit in chronically anaemic adult patients at Kimberley Hospital Complex, South Africa,^9^ assessed whether medical practitioners transfused according to the American Association of Blood Banks (AABB) transfusion guidelines,^10^ which have recently been updated^11^ and recommend transfusion when a patient has a haemoglobin level of < 6.9 g/dL and symptoms of anaemia.^10,11^ The Kimberley Hospital adhered to transfusion guidelines and most transfusions (76%) were appropriate.^9^

Restrictive transfusion, where a patient is transfused at a haemoglobin level of < 8 g/dL, is safe and yields good outcomes even in older patients.^12,13^ Transfusing RBCs to anaemic or bleeding patients aims to restore oxygen-carrying capacity, thus improving tissue oxygenation.^1^ It also expands circulating volume, especially in bleeding patients.^6,13^ Red blood cell transfusion does not restore oxygen-carrying capacity in critically ill patients and is associated with poorer patient outcomes.^2^

Practitioners should perform proper and thorough investigations on all anaemic patients to identify and manage the primary cause instead of simply transfusing the patient with the cause remaining unknown.^1^ Frail patients should receive transfusions only if they have severe symptoms and when other treatment modalities, such as iron therapy, among others, have not been effective.^1^

Anaemic patients booked for elective surgical procedures must be managed by adequately investigating the underlying causes and ensuring that the patient’s haemoglobin is optimised based on the definitive cause before the surgical procedure. Patients who undergo emergency procedures with haemoglobin values of < 8 g/dL need to be transfused.^1^ Obstetric haemorrhage is a life-threatening emergency, and the patient needs transfusion when the haemoglobin is between 6 g/dL and 10 g/dL or when the patient has symptoms of inadequate perfusion, such as heart failure and dyspnoea at rest.^1^

Transfusion of RBCs is associated with several risks, including transfusion reactions.^1^ Patient safety is paramount. To minimise these risks, checklists and other procedures that practitioners should follow before and during transfusion, are available. These include patient identification and verification, valid patient consent, blood component identification and verification, patient monitoring, identification and prompt management of adverse reactions.^1,2,13,14^

The American College of Pathologists audited RBC transfusions in 128 United States hospitals to assess compliance with institutional guidelines,^15^ focusing on haemoglobin as a transfusion trigger. A median haemoglobin threshold of 8.0 g/dL – 8.9 g/dL for patients of all categories (e.g., pre-operative, intraoperative with bleeding, postoperative with bleeding, critically ill patients, haematology patients, oncology patients) was used, except for patients with cardiovascular and respiratory disease, for whom a limit of 9.0 g/dL – 9.9 g/dL was applied. Of all the hospitals that participated in the study, 69% were found to be compliant.^15^ More developed countries are moving towards evidence-based practice, such as transfusing at lower haemoglobin levels and increasing single-unit transfusion, as indicated in a study conducted in Finland.^16^

Clinical audits and studies in obstetrics and gynaecology indicated that patients were transfused inappropriately, as evidenced by transfusing at haemoglobin levels above the threshold and exceeding the targeted haemoglobin levels leading to overtransfusion.^17,18^ A study in Bangladesh found that 9.23% of transfusions in patients admitted to obstetrics and gynaecology were unnecessary.^18^ Sixty-four per cent of the patients had pre-operative anaemia, and 24% had antenatal anaemia, both treatable causes; 46% received transfusions secondary to miscarriages. Interventions such as early diagnosis of anaemia and initiation of iron therapy were sometimes delayed or inadequate.^18^ Evidence shows that a clinical audit is one of the most valuable instruments to determine whether transfusion practices align with the guidelines and identify knowledge deficiencies.^7,19,20^

The National District Hospital (NDH) is a Level-1 hospital in Bloemfontein, South Africa. The various service points are the maternity ward, male wards, female wards, paediatric ward, day ward (surgical), two theatres, emergency unit, outpatient clinic and a crisis centre. The emergency unit is open 24 h a day. Theatre hours are Monday to Friday from 08:00 till 16:00. Surgical procedures offered include caesarean sections, minor surgeries, uterine evacuations and sterilisations. The average daily bed occupancy cost is $236.00.

The hospital is situated 3.9 km from Universitas Academic Hospital, a central hospital providing the most highly specialised level of care in the Free State province. The blood bank servicing NDH is at Universitas Academic Hospital. One unit of RBCs is available on-site for emergency transfusion purposes at any given time.

The study aimed to conduct a clinical audit of RBC transfusion practices at a district hospital in South Africa. The study’s objectives were to:

- describe the current practice of RBC transfusion at NDH
- identify clinical characteristics and demographic profiles of the patients receiving RBC transfusions
- determine current transfusion triggers and correlate them with the clinical guidelines for the use of blood products in South Africa^1^
- evaluate post-transfusion outcomes of patients at NDH
- determine adherence to the clinical guidelines for the use of the blood products in South Africa.^1^

## Methodology

### Study design

A retrospective descriptive study was conducted.

### Study population and sample

The study population included all patients at all service points who received a blood transfusion for any clinical condition at NDH between 01 June 2019 and 31 December 2019. Patients with missing files and incomplete information were excluded from the study. A total of 76 transfusion episodes performed on 72 patients were reviewed.

### Data collection

All blood transfusion registers at NDH were used to identify all transfusion episodes. For the purposes of this study, a transfusion episode was defined as one or more units of RBCs transfused from a single order. Patient files were retrieved from the admissions office using the transfusions listed in the registers. Each patient was allocated a study number used on the datasheet.

Data were captured on a data sheet created based on the literature and study objectives. The data sheet comprised the following five sections:

- Section A: demographic information, diagnosis and comorbid conditions.
- Section B: pre-existing anaemia, indication for transfusion, workup and additional treatment.
- Section C: signed consent, presence of two verification signatures.
- Section D: pre- and post-transfusion haemoglobin levels recorded, number of units ordered, transfused and/or returned, transfusion time, baseline, during and post-transfusion vital signs recorded, transfusion reactions.
- Section E: adherence to guidelines, patient outcome, 30 days follow-up outcome.

We evaluated the appropriateness of the transfusion based on adherence to the following criteria as specified in the South African transfusion guidelines^1^:

- pre-transfusion haemoglobin level
- comorbid conditions
- investigations for anaemia workup, additional treatment
- presence of valid consent, two verification signatures
- transfusion time of less than 6 h
- documentation of post-transfusion haemoglobin level, baseline, during transfusion and post-transfusion vital signs
- record of transfusion reactions.

A transfusion was considered adherent to the guidelines when all the criteria were met, that is, all the items on the list needed to be documented, the transfusion time needed to be documented and should be less than 6 h.

### Data analysis

The information from the datasheet was captured in a Microsoft Excel (version 2016) spreadsheet. The Department of Biostatistics, Faculty of Health Sciences, University of the Free State, performed the statistical analysis, using Statistical Analysis Software (SAS 9.4). Numerical variables were summarised by medians, minimum, maximum, or percentiles. Categorical variables were summarised by frequencies and percentages. Differences between pre- and post-transfusion groups were evaluated using the signed rank test for paired data.

### Ethical considerations

The study was approved by the Health Sciences Research Ethics Committee (HSREC) of the University of the Free State (UFS-HSD2020/1109/2508). Permission to conduct the study was obtained from the Free State Province Department of Health. All patient-related information was kept confidential, and no identifiable patient details were used in any form. Because of the retrospective nature of the study and using transfusion registers and patient files to obtain information, no informed consent from the patients was required.

## Results

### Patients’ demographic and clinical information

**FIGURE 1:**
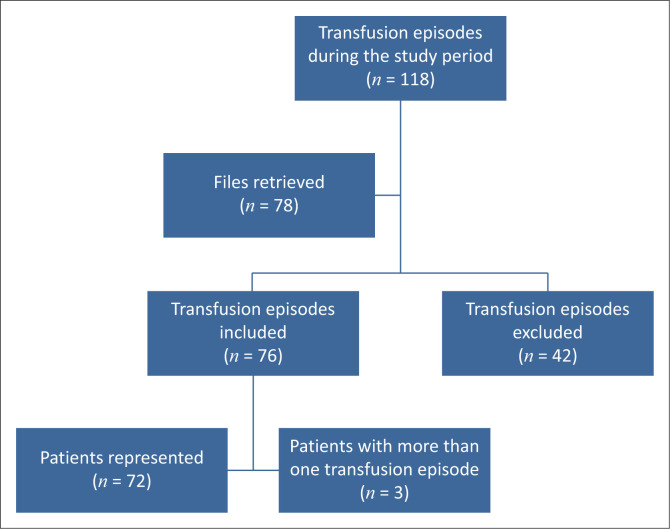
Transfusion episodes and patient numbers.

As illustrated by Figure 1, the 76 episodes that met the transfusion criteria represented 72 patients – two patients had two separate transfusion episodes each and one patient had three transfusion episodes. Each transfusion episode was captured on a separate data sheet.

Most patients were females (*n* = 54; 75.0%). The median age was 47 years (range 13–89 years, interquartile range [IQR]: 32–66 years). Table 1 summarises the patients’ admission diagnosis and comorbidities. Anaemia was the most common admission diagnosis (25.0%), followed by pneumonia (19.4%) and renal failure (13.9%). Under the admission diagnoses categorised as ‘Other’, the most common were delirium (4.2%), peptic ulcer disease (PUD) (2.8%) and postpartum haemorrhage (PPH) (1.4%). The most common comorbidities were HIV infection (44.4%) and hypertension (33.3%). Other underlying chronic medical conditions were also identified, such as pulmonary tuberculosis (4.2%), venous ulcer (2.8%) and angioectasis, asthma, abnormal uterine bleed, cataract, COPD, epilepsy, hypothyroidism, iron deficiency anaemia, myelofibrosis, PUD, valvular disease, fibroids and rectal bleed, all at 1.4%, respectively.

**TABLE 1:** Clinical characteristics of the patients (*n* = 72).

Clinical characteristics	*n*	%
**Admission diagnosis** †		
Anaemia	18	25.0
Pneumonia	14	19.4
Renal failure	10	13.9
Abnormal uterine bleed	9	12.5
Abortion	4	5.6
Severe epistaxis	3	4.2
Gastrointestinal bleed	3	4.2
Malignancy	3	4.2
Caesarean section	2	2.8
Congestive cardiac failure	2	2.8
Other	26	36.1
**Comorbid conditions** ‡		
HIV infection	32	44.4
Hypertension	24	33.3
Diabetes	8	11.1
Chronic kidney disease	4	5.6
Malignancy	4	5.6
Congestive cardiac failure	3	4.2
Other	19	26.4

† , Patients could have more than one admission diagnosis;

‡ , Patient could have more than one comorbidity.

Only 18 (25.0%) of the 72 patients had pre-existing anaemia, of which 14 (19.4%) patients presented with iron deficiency, two (2.8%) with anaemia of inflammation, and one (1.4%) patient each with vitamin B12 deficiency and myelofibrosis, respectively. In almost all of the transfusion episodes (*n* = 75/76; 98.7%), the indication for transfusion was symptomatic anaemia. One patient (1.4%) was preoperative.

Table 2 highlights the various investigations performed and additional treatment given. Oral iron therapy was the most commonly prescribed treatment (*n* = 62; 81.6%). In 76.3% (*n* = 58) of the transfusions, folate was given as an additional treatment. No patient received erythropoietin, while a small percentage received the antifibrinolytic agent tranexamic acid (*n* = 12; 15.8%) and vitamin B12 (*n* = 2; 2.6%).

**TABLE 2:** Anaemia investigations and additional treatment (*n* = 76).

Variables	*n*	%
**Anaemia investigations**		
Iron studies	51	67.1
Folate	45	59.2
Vitamin B12	44	57.9
Reticulocyte production index	26	34.2
Haptoglobin	10	13.2
**Additional treatment**		
Oral iron	62	81.6
Folate	58	76.3
Tranexamic acid	12	15.8
Vitamin B12	2	2.6
Erythropoietin	0	0

A patient’s pre-transfusion haemoglobin level was one of the transfusion triggers and was documented for all 76 (100%) transfusions. The median haemoglobin level was 4.6 g/dL (range: 2.5 g/dL – 9.5 g/dL, IQR: 3.95 g/dL – 5.5 g/dL). Post-transfusion haemoglobin level was recorded for 55 (72.4%) transfusions, with the median being 6.9 g/dL (range: 3.0 g/dL – 11.0 g/dL, IQR: 5.4 g/dL – 7.9 g/dL). The median difference between the pre- and post-transfusion haemoglobin levels as calculated using the signed rank test was statistically significant (*p* < 0.0001).

### Monitoring

A single unit of RBC was ordered for 52.6% (*n* = 40) of the transfusion episodes. Two units were ordered for 46.1% (*n* = 35) of the transfusion episodes, and three units were ordered for one (1.3%) transfusion episode. Patients with an admission diagnosis of malignancy were the only ones who received a single unit. The rest were transfused two or more units. All units ordered were transfused and none returned to the blood bank. The median transfusion time was 6.5 h (range: < 1 h – 16 h, IQR: 4.0–9.0 h).

Baseline vital signs were recorded in 100% of transfusions. regular vital signs were recorded during the transfusion in 75 (98.7%) episodes, while 71 (93.4%) of the transfusions had post-transfusion vital signs recorded. All 76 transfusion episodes audited contained signed consent and two verification signatures.

### Adherence to guidelines

**FIGURE 2:**
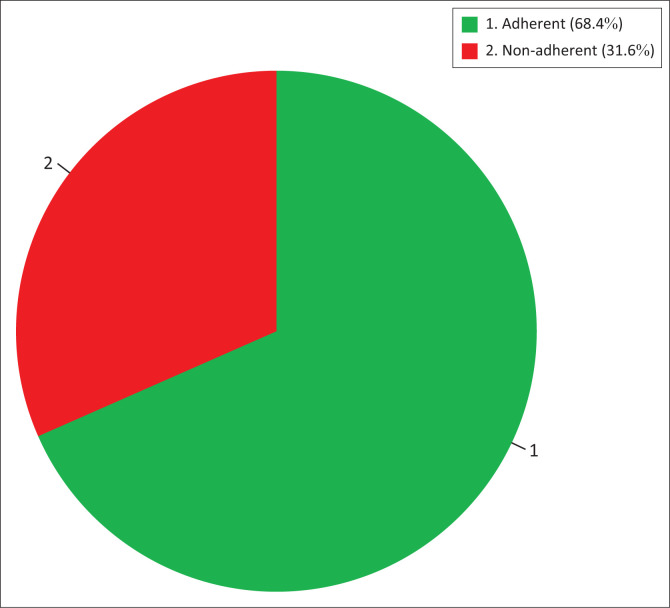
Adherence to the clinical guidelines for the use of blood products in South Africa^1^ during red blood cell transfusions (*n* = 76).

Figure 2 illustrates the frequency of adherence to the clinical guidelines for the use of blood products in South Africa.

### Patient outcomes

Only one patient developed a documented transfusion reaction presenting as chest pain. In 72 (94.7%) of the 76 transfusion episodes, the patients were discharged and for 71 (93.4%) transfusions, patients had referral letters for follow-up visits either at the hospital or local clinic. No death was recorded in the study. Three patients were transferred from NDH to other facilities.

For patient outcomes at the 30-day follow-up visit, data for more than half of the transfusion episodes (*n* = 56, 73.7%) were missing. Patients of five (6.6%) transfusion episodes were re-admitted. Patients of 14 (18.4%) transfusion episodes were recorded as healthy. No deaths were reported at follow-up.

## Discussion

Blood transfusion is one of the most expensive life-saving treatments in clinicians’ daily practices. However, there is an increasing trend towards a more restrictive approach.^2,13,15^ More than 80 units of RBCs were transfused, costing NDH more than $10 525.00 in total, representing an average cost of approximately $132.00 per unit (personal communication, 16 March 2020, Chief Executive Officer of NDH). The absence of a blood bank on the hospital premises and the limited emergency blood supply served as motivation to investigate whether patients at NDH are assessed for primary causes of anaemia appropriately, and whether alternative treatments are used as recommended by the clinical guidelines for the use of blood products in South Africa.^1^

The median age was 47 years, aligned with similar studies reporting a mean age of 42 years and 47.3 years, respectively.^3,8^ The sex distribution of patients in our study, with a female to male ratio of 3:1, was similar to previously reported findings.^3,12,21^

Anaemia was the most common admission diagnosis. However, anaemia is a symptom of an underlying cause that needs to be investigated in order to make an appropriate diagnosis.^1^ Pneumonia was the second-highest admission diagnosis among the patients.

Evidence shows that RBC transfusion predisposes a patient to acute respiratory distress syndrome.^2,13^ Moreover, the risk of morbidity and mortality is higher in critically ill patients.^2^ Therefore, every patient should be individualised with regard to age, comorbidities and severity of the existing illness. Furthermore, the risks associated with blood transfusion, such as volume overload and infections, among others, versus the benefit of eradicating the symptoms of blood loss or anaemia, should be considered before deciding to transfuse.^13^

Patients with ongoing blood loss are occasionally transfused as part of resuscitation, such as acute gastrointestinal bleeding or severe epistaxis, which, respectively, occurred in 4.2% of the patients in the admission diagnosis category of our study. These patients can also be managed with additional treatment such as tranexamic acid.^1^

In this study, more than 40% of the patients were HIV-positive. A high percentage was expected because of the prevalence of HIV in South Africa, estimated at 13.9% in 2022.^22^ HIV predisposes patients to different types of cytopenia and anaemia of inflammation.^1,3,6,7^ Subsequently, many of them will present to the hospital with symptomatic anaemia. It appears that these patients get transfused despite associated risks and the availability of alternative treatment. An anaemia workup should be performed for all HIV-positive patients as they may have other causes of anaemia and not necessarily cytopenia.

A third of the patients in this study had hypertension, which can lead to complications such as renal impairment, congestive cardiac failure and end-diastolic dysfunction, all risk factors for transfusion-associated circulatory overload.^14^ Hypertension is also one of the transfusion-related adverse reactions.^1^ There should be proper, regular monitoring before, during and post transfusions. For example, furosemide should be prescribed to patients who are at risk of transfusion-associated circulatory overload.^23^ However, its use was not part of the study audit. Workup to determine the specific cause of the patient’s anaemia is of critical importance, as pointed out in the principles of patient blood management.^6,21^ Most of the patients in our study were investigated for underlying causes, which included iron studies (67.1%) and folate (59.2%).

Research has suggested that most patients with anaemia could be managed with alternative therapies based on their anaemia workup results.^1,24^ Oral iron supplementation was administered during 81.6% of the transfusion episodes. Unfortunately, parenteral iron was not available at the district hospital level in the Free State province during the study period. Consequently, patients received RBC transfusion instead of iron therapy only. This resulted in serious cost implications on the healthcare system as the approximate cost of iron dextran–hydroxide dextran complex 50 mg/mL, 10 mL is $17.00 compared to $104.00 for a single RBC unit (Formulary March 2024, Free State Province Department of Health).

Folate therapy was also generously used (76.3%). Yet, it is of concern that only 15.8% of the patients received tranexamic acid despite 26 patients presenting with a history of acute blood loss and strong evidence confirming that tranexamic acid limits blood loss.^25^ No patient received erythropoietin despite its benefit in patients with renal failure,^1^ which was the admission diagnosis in 13.9% of transfusion episodes. The reason could be that erythropoietin could not be initiated at district hospital level at the time of the study. Only two patients received vitamin B12, which was tested in 57.9% of transfusion episodes.

Symptomatic anaemia was the indication for transfusion in almost all patients in this study. The pre-transfusion haemoglobin was documented for all transfusion episodes. The median haemoglobin was 4.6 g/dL. A similar study in Kimberley reported that in 96% of patients, the transfusion trigger was symptomatic anaemia, with a median haemoglobin value of 6.2 g/dL.^9^ Our finding on haemoglobin levels was notably different from the American College of Pathologist study, which had a higher pre-transfusion median haemoglobin of 8.1 g/dL.^16^ Nevertheless, it is essential to note that haemoglobin value is not the only determining factor for transfusing a patient. Patients’ comorbidities and clinical status are also vital factors.^1^ Thus, patients in this audit were transfused at considerably lower haemoglobin values than in other studies.

The files of all transfusion episodes had a valid signed consent document as well as two verification signatures. In more than half of the transfusions, patients had a single unit of blood ordered, in keeping with the World Health Organization’s recommended patient blood management principles.^5^ The difference may be because of the pre-transfusion haemoglobin in that study being higher with a median of 6.2 g/dL. It could be concluded that the patients benefited from transfusion considering that the post-transfusion haemoglobin was significantly higher than the pre-transfusion (median level 4.6 g/dL) (*p* < 0.0001).

Clinical guidelines for the use of blood products in South Africa^1^ recommend that blood must be transfused within 6 h of starting the transfusion. We found a median transfusion time of 6.5 h, which means by implication that more than 50% of the transfusions exceeded 6 h.

Literature indicates that baseline vital signs should be noted before initiating a transfusion, closely monitored for the initial 30 min, then every 30 min for the duration of the transfusion if the patient is not bleeding serverly, in which case monitoring should be done every 15 min. Monitoring should continue for 12 h to 24 h post-transfusion. Documentation showed that monitoring of pre-transfusion vital signs was good. Almost all patients’ vital signs were adequately monitored during the transfusions. However, there was a decline in the documentation post-transfusion as vital signs were recorded afterwards in 93% of episodes, compared to 99% during transfusion. This practice could compromise patients’ safety, as complications, for example, transfusion-related lung injury and transfusion-associated circulatory overload, might not be detected timeously, resulting in poor patient outcomes.^1^

Only one case of an adverse effect was reported during the study period when a patient developed chest pains. It might be that adverse effects do not commonly occur at NDH as seen in a study conducted in Ethiopia that found that only 5.2% of patients developed adverse effects.^21^ Almost all patients were referred to other facilities or departments for continued care or further investigations. Only three patients were transferred to other facilities as inpatients. The majority of patients were discharged home and received referral letters for follow-up at other facilities as outpatients. No deaths were recorded.

Collecting data for the follow-up visits at 30 days was challenging as no information could be found for 75% of the transfusion episodes. The reason could be that patients were referred to other facilities for continued care. The patients of five transfusion episodes were re-admitted and 18.4% were noted as healthy.

This study described transfusion practices and evaluated adherence to the clinical guidelines for the use of blood products in South Africa^1^ at NDH. The audit revealed that 68% of the transfusion episodes adhered to the guidelines (see Figure 2). Improvement in areas such as record keeping, anaemia workup for anaemic patients who are not bleeding, additional treatment such as iron and folate therapy to confirmed iron and folate-deficient patients, and monitoring and documentation of post-transfusion vital signs, could be achieved through continuous training and repeat audits, as demonstrated by similar studies.^9,26^

The study went into great depth to describe the transfusion practices at NDH. The audit tool was extensive, thus obtaining substantial relevant patient information. On the other hand, the sample size was small because of few transfusion episodes at the hospital during the study period and difficulties in retrieving files. Missing and incomplete records were also a limiting factor in this study. Therefore, these pose a challenge in generalising the findings of the study to other settings. Different hospitals can create their own audit tools based on the current transfusion guidelines for their specific context to monitor their transfusion practices and keep revising them to ensure that they remain relevant to the latest literature.

### Recommendations

Based on the findings of this study, the authors propose the following recommendations:

- A clear, detailed transfusion protocol relevant to a district hospital and patient profile should be written.
- Regular training on patient blood management is a necessity.
- Periodic quality improvement cycles regarding transfusion practices should be conducted.
- Patients with acute blood loss and those undergoing surgical procedures with a high risk of blood loss should receive tranexamic acid.
- Transfusion personnel should monitor the transfusion rate and limit the duration to 6 h maximum. All transfusion-related adverse reactions should be anticipated based on the patient’s clinical status and underlying medical condition and documented.

A submission will be made to the provincial Drugs and Therapeutics Committee to include erythropoietin in the drug schedule for district hospitals to treat anaemia. Currently, it is only available to treat anaemia at Universitas Academic Hospital, a tertiary level hospital, with code restrictions for nephrology patients and in high doses for oncology patients with bone marrow suppression.

This recommendation for wider access can be justified by the significant costs for a single RBC unit (> $104.00), compared to the erythropoietin prefilled syringe (approximately $2.60 per 2000 IU/0.5 mL prefilled syringe, with 1–3 injections per week needed) (FS Formulary March 2024, Free State Province Department of Health).

## Conclusion

This study demonstrated that most transfusion episodes at NDH adhered to the clinical guidelines for the use of blood products in South Africa. However, there were still some gaps regarding alternative treatment and monitoring post-transfusion. Future training of personnel might lead to improvements in practice followed by more audits.
